# BD-KDD: A real-world clinical dataset for kidney disease diagnosis and healthy classification

**DOI:** 10.1016/j.dib.2026.112738

**Published:** 2026-03-30

**Authors:** Muhammad Towfiqur Rahman, Salma Akter, Md. Masudul Islam, Md. Shafiqul Islam

**Affiliations:** a Bangladesh University of Business and Technology, Dhaka, Bangladesh; b University of Asia Pacific, Dhaka, Bangladesh

**Keywords:** Kidney disease dataset, Renal biomarkers, Chronic nephropathy, Biomedical records, Predictive modelling, Health informatics, Medical classification

## Abstract

Kidney disease is a major global health concern that requires timely diagnosis and effective monitoring to prevent severe complications and improve patient outcomes. This data article presents BD-KDD, a structured clinical dataset designed to facilitate research on kidney disease diagnosis. The dataset was collected retrospectively from electronic medical records obtained from Popular Diagnostic Center, Savar Branch, Dhaka, Bangladesh, following institutional authorization for academic research. The BD-KDD dataset contains 988 patient records with 26 variables, including demographic attributes, physiological measurements, biochemical laboratory tests, urinalysis indicators, hematological parameters, comorbidity indicators, and clinical symptoms. Key laboratory features include serum creatinine, blood urea, blood glucose, sodium, potassium, hemoglobin, packed cell volume, red blood cell count, and white blood cell count, along with urinalysis indicators such as specific gravity, albumin, and sugar levels. Each record is assigned a binary diagnostic label representing either healthy individuals or kidney disease cases based on clinical evaluation and laboratory findings. The curated dataset includes 481 healthy and 507 kidney disease cases and is provided in CSV format together with a dataset dictionary describing variable definitions and coding schemes. BD-KDD offers a valuable resource for biomedical data analysis, health informatics research, and the development of machine learning–based diagnostic models and clinical decision support systems for renal health assessment.

Specifications Table

**Table:** 

Subject	Computer Sciences
Specific subject area	Clinical nephrology and biomedical informatics focusing on structured laboratory records for renal health assessment and disease classification
Type of data	Table, Raw
Data collection	Clinical laboratory and demographic records of 988 individuals were retrospectively collected from the electronic medical records of Popular Diagnostic Center, Savar Branch, Dhaka, Bangladesh, following official authorization for academic research. Measurements including blood pressure, serum creatinine, urea, glucose, sodium, potassium, hemoglobin, RBC, and WBC counts were obtained using automated clinical chemistry and hematology analyzers under routine diagnostic protocols. Patients were labeled as kidney disease or healthy based on physician evaluation and laboratory reports. All records were anonymized, screened for completeness, and normalized before analysis.
Data source location	The data were collected from Popular Diagnostic Center, Savar Branch, Dhaka, Bangladesh, located in Talbagh, Anandapur, Savar (approx. 23.858° N, 90.267° E). The dataset consists of anonymized patient laboratory and clinical records obtained from the center’s electronic medical records for research purposes with institutional authorization.
Data accessibility	Repository name: **Harvard Dataverse** Data identification number: https://doi.org/10.7910/DVN/MB1LES Direct URL to data: https://dataverse.harvard.edu/dataset.xhtml?persistentId=doi:10.7910/DVN/MB1LES **Instructions for accessing these data:** To access the BD KDD clinical dataset for kidney disease diagnosis, navigate to its Harvard Dataverse repository using the provided link. Once on the main page, scroll down to the Files section, which contains a total data volume of approximately one point eight megabytes. You will find three specific files available for open use. To acquire the actual records, click the Download button next to the BD KDD Dataset csv file. Additionally, download the BD KDD Dictionary md file to clearly understand the twenty-six dataset variables. If you prefer to get everything at once, simply click the Download all button.
Related research article	NA

## 1 Value of the Data

• The dataset contains clinical and laboratory records of 988 individuals, including both healthy and kidney disease cases, providing a structured resource for studying renal health indicators and associated biomedical variables.
• The data include multiple routinely measured clinical biomarkers such as blood pressure, serum creatinine, urea, glucose, sodium, potassium, hemoglobin, RBC, and WBC counts, enabling researchers to examine relationships between laboratory parameters and renal conditions.
• The data are cleaned, anonymized, and organized in a structured tabular format, facilitating reuse for biomedical data analysis and enabling researchers in biomedical informatics, medical data science, and artificial intelligence in healthcare to develop predictive models and clinical decision-support tools.
• Researchers can reuse these data to develop, benchmark, and compare machine learning or statistical models for medical classification, prediction, and risk assessment tasks related to renal conditions.
• The dataset can also support methodological studies in biomedical data preprocessing, feature selection, and predictive modelling, particularly in research focusing on clinical decision support systems and healthcare analytics.

## 2 Background

Kidney disease is a serious global health problem that affects millions of individuals and can lead to severe complications such as cardiovascular disorders, metabolic imbalance, and organ failure if not detected and managed early [1]. Clinical diagnosis and monitoring of renal disorders rely heavily on laboratory biomarkers and physiological indicators. Measurements such as serum creatinine, blood urea, electrolyte concentrations, blood pressure, and hematological parameters provide essential information about kidney function and are routinely used in nephrology to evaluate renal health and disease progression [2,3]. The rapid growth of electronic medical records and digital healthcare systems has created new opportunities for applying data-driven techniques in medical research. In recent years, machine learning and statistical modelling approaches have been increasingly used to analyze clinical data, identify disease patterns, and support clinical decision-making. The development and validation of such computational methods require access to well-structured clinical datasets that contain relevant laboratory and patient health information. However, publicly available datasets for kidney disease research remain relatively limited. One commonly used resource is the Chronic Kidney Disease dataset from the UCI Machine Learning Repository, which contains 400 patient records with several clinical attributes related to renal diagnosis and has been widely used for machine learning classification studies [4]. Despite its usefulness, the limited size and variability of existing datasets highlight the need for additional structured clinical data resources. To address this need, the BD-KDD dataset was compiled from routine diagnostic laboratory records obtained from a clinical diagnostic center in Dhaka, Bangladesh. The dataset contains anonymized clinical and laboratory measurements associated with renal health and provides structured information that can support biomedical data analysis, predictive modelling, and research on data-driven approaches for kidney disease diagnosis.

## 3 Data Description

**Fig. 1:**
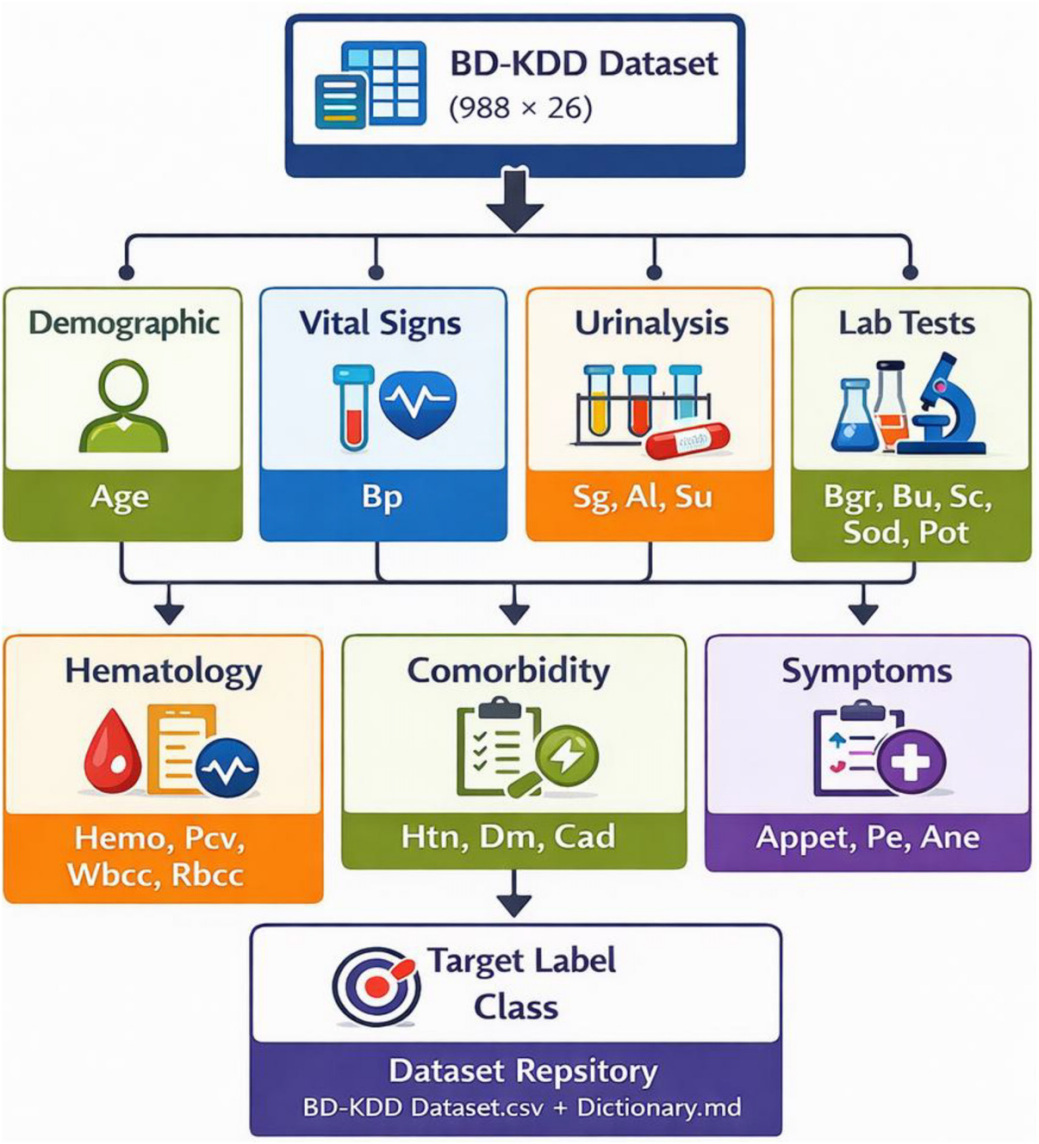
Category grouping of variables included in the BD-KDD dataset.

**Fig. 2:**
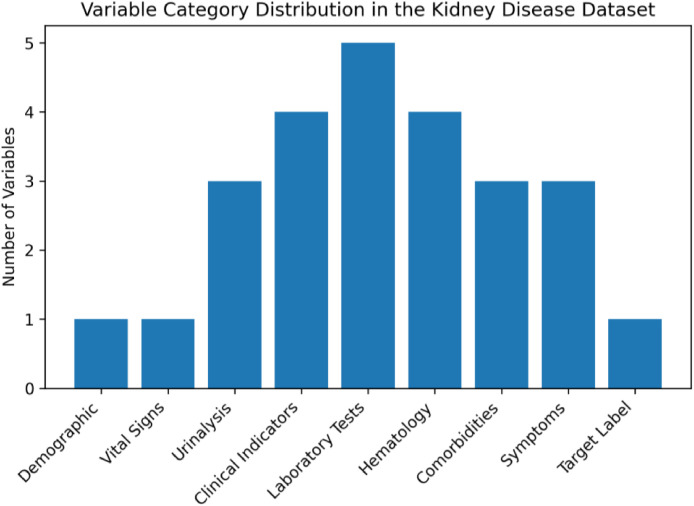
Variable category distribution in the kidney disease dataset.

**Fig. 3:**
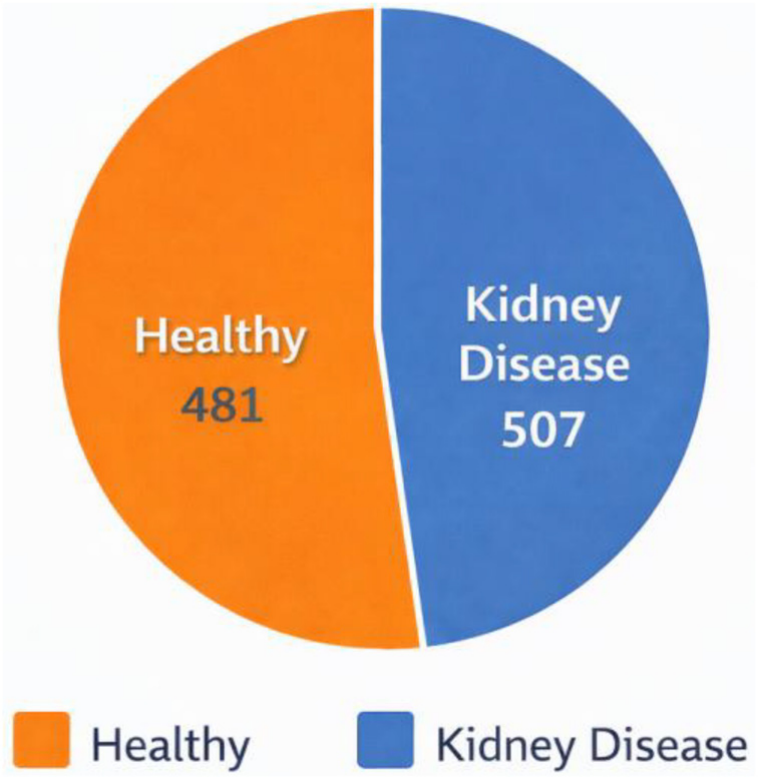
Distribution of diagnostic classes in the BD-KDD dataset.

The dataset is provided as a structured repository containing tabular clinical records related to renal health assessment. The dataset stored in public repository called Harvard Dataverse [5] which includes one primary dataset file organized in comma-separated value format. The dataset contains 2 files: (i) BD-KDD Dataset.csv which is main dataset file and (ii) BD-KDD Dictionary.md which is dataset dictionary file. The primary dataset file contains 988 rows representing individual patient records and 26 columns representing clinical variables and diagnostic labels. Table 1 provides a summary of the variables included in the dataset and Fig. 1 illustrates the Category grouping of variables included in the BD-KDD dataset. The dataset includes both demographic and biochemical laboratory indicators commonly used in nephrology diagnostics. Variables include physiological measurements (blood pressure), biochemical laboratory tests (creatinine, urea, glucose), hematological parameters, and binary indicators describing comorbid conditions or symptoms. All patient identifiers were anonymized and represented using coded IDs. Each row corresponds to a single patient record. The dataset variables are grouped into demographic, vital signs, urinalysis indicators, clinical indicators, laboratory tests, hematology measurements, comorbidities, symptoms, and the diagnostic class label which is represented in Table 2 and Fig. 2. A class distribution chart is showing the healthy and disease class count in Fig. 3.

**Table 1:** Overview of dataset variables.

Variable	Description	Data Type	Possible Values / Range
Sl. No.	Serial Number	Numeric	1,2,3,4…988
Age	Patient age	Numeric	Years
Bp	Blood pressure	Numeric	mmHg
Sg	Specific gravity (urine)	Numeric	1.005–1.025
Al	Albumin level	Numeric	0–5
Su	Sugar level	Numeric	0–5
Rbc	Red blood cells presence	Binary	0 = abnormal, 1 = normal
Pc	Pus cell presence	Binary	0 = abnormal, 1 = normal
Pcc	Pus cell clumps	Binary	0 = no, 1 = yes
Ba	Bacteria presence	Binary	0 = no, 1 = yes
Bgr	Blood glucose random	Numeric	mg/dL
Bu	Blood urea	Numeric	mg/dL
Sc	Serum creatinine	Numeric	mg/dL
Sod	Sodium	Numeric	mEq/L
Pot	Potassium	Numeric	mEq/L
Hemo	Hemoglobin	Numeric	g/dL
Pcv	Packed cell volume	Numeric	%
Wbcc	White blood cell count	Numeric	cells/cumm
Rbcc	Red blood cell count	Numeric	million cells/cumm
Htn	Hypertension status	Binary	0 = no, 1 = yes
Dm	Diabetes mellitus	Binary	0 = no, 1 = yes
Cad	Coronary artery disease	Binary	0 = no, 1 = yes
Appet	Appetite condition	Binary	0 = poor, 1 = good
Pe	Pedal edema	Binary	0 = no, 1 = yes
Ane	Anemia	Binary	0 = no, 1 = yes
Class	Diagnostic class label	Binary	0 = healthy, 1 = kidney disease

**Table 2:** Variable category summary.

Category	Variables
Demographic	Age
Vital Signs	Blood Pressure
Urinalysis	Sg, Al, Su
Laboratory Tests	Bgr, Bu, Sc, Sod, Pot, Hemo
Hematology	Pcv, Wbcc, Rbcc
Clinical Indicators	Rbc, Pc, Pcc, Ba
Comorbidities	Htn, Dm, Cad
Symptoms	Appet, Pe, Ane
Target Label	Class

A descriptive statistical analysis was performed to provide an overview of the dataset distribution and quality. The dataset contains 988 patient records, with 507 kidney disease cases (51.3%) and 481 healthy individuals (48.7%), indicating a balanced class distribution suitable for classification tasks. Numerical variables such as age, blood pressure, serum creatinine, blood urea, and hemoglobin exhibit clinically realistic ranges consistent with standard nephrology references. The dataset was screened for missing and inconsistent values during preprocessing. Records with incomplete or contradictory clinical information were excluded, resulting in a clean dataset with minimal missing values. Binary variables (e.g., hypertension, diabetes mellitus, anemia) were encoded consistently as 0 and 1. Preliminary analysis shows that key biomarkers such as serum creatinine, blood urea, and hemoglobin demonstrate distinguishable patterns between healthy and diseased groups, supporting their relevance in predictive modelling. This statistical overview confirms the dataset’s internal consistency and suitability for data-driven kidney disease research.

## 4 Experimental Design, Materials and Methods

**Fig. 4:**
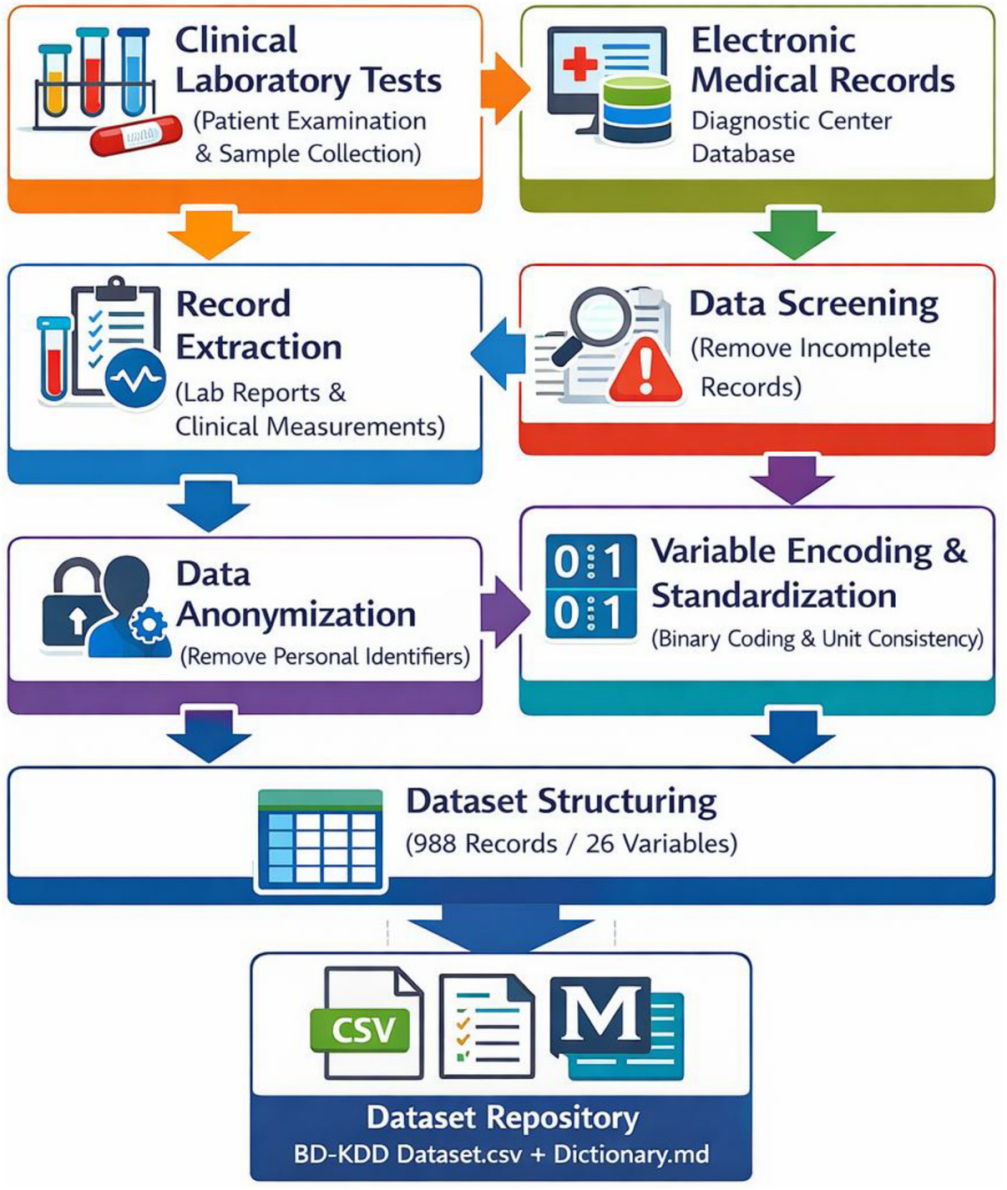
Clinical dataset acquisition and preparation workflow for Kidney Disease Diagnosis.

The BD-KDD dataset was compiled through a structured retrospective data acquisition and curation process designed to extract clinically relevant laboratory and physiological measurements related to renal health. The data were obtained from the electronic medical record (EMR) system of a diagnostic laboratory after receiving institutional authorization for academic research use. Only anonymized clinical records were included in the dataset. The acquisition procedure consisted of four primary stages: clinical record extraction, laboratory measurement documentation, data cleaning and anonymization, and dataset structuring for research reuse. The dataset includes patient records that met the following inclusion criteria: (i) availability of complete laboratory test results relevant to renal function assessment, (ii) presence of key biochemical and hematological measurements, and (iii) clear diagnostic labeling based on physician evaluation. Records were excluded if they contained missing critical laboratory values, inconsistent or contradictory clinical information, duplicate entries, or incomplete diagnostic reports. Only records with sufficient clinical evidence to support classification as either healthy or kidney disease were retained. Fig. 4. represents workflow diagram and detailed description outline the comprehensive process used to collect and organize the BD-KDD kidney diagnosis dataset. Initially, patient laboratory reports and associated clinical measurements were retrieved from the diagnostic center’s electronic database. These records correspond to routine diagnostic tests performed under standard laboratory protocols. Laboratory measurements were generated using automated clinical chemistry analyzers and hematology analyzers commonly used in diagnostic laboratories to quantify biochemical and hematological parameters. Measurements included serum biomarkers, electrolyte concentrations, hematological indicators, urinalysis observations, and physiological vital signs. Blood samples were processed using automated chemistry analyzers to obtain biochemical parameters such as serum creatinine, blood urea, blood glucose, sodium, potassium, and hemoglobin levels. Hematological measurements including packed cell volume, red blood cell counts, and white blood cell counts were obtained through automated hematology analyzers. Urinalysis indicators including specific gravity, albumin, sugar, and microscopic observations such as red blood cells, pus cells, and bacteria presence were recorded based on standard diagnostic laboratory examination procedures. Blood pressure values were documented from routine clinical measurements recorded during patient examination.

Following data acquisition, all records were screened to remove incomplete or inconsistent entries. Personal identifiers such as patient names, addresses, or hospital registration numbers were removed to ensure full anonymization and compliance with privacy requirements. Each patient entry was assigned a coded serial identifier to maintain record uniqueness while preventing identification of individuals. Binary clinical indicators were standardized using numerical encoding where categorical states were represented as 0 or 1 values. Numerical laboratory measurements were retained in their original measurement units as recorded in the clinical reports. The curated records were then organized into a structured tabular dataset consisting of 988 rows representing individual patient observations and 26 columns representing clinical attributes and the diagnostic class label. The dataset variables were grouped according to measurement type, including demographic attributes, vital signs, urinalysis indicators, clinical observations, biochemical laboratory tests, hematological measurements, comorbidity indicators, and clinical symptom indicators. The diagnostic class label was assigned based on physician evaluation documented in the medical reports and laboratory findings.

Data organization and preprocessing were conducted using commonly available data processing tools. The dataset was compiled and structured using spreadsheet-based data handling software and subsequently exported to a comma-separated values (CSV) format to facilitate platform-independent access and reproducibility. The dataset dictionary file describing each variable, coding scheme, and measurement unit was prepared using a structured markdown document to support interpretation and reuse of the dataset. Basic verification and formatting procedures were performed using standard data analysis libraries within the Python programming environment to ensure consistent encoding and formatting across all records. The resulting dataset repository contains two primary files: the main structured dataset file in CSV format and a dataset dictionary file describing the variables and coding scheme. This structure enables straightforward reuse for statistical analysis, biomedical data mining, and machine learning research without requiring additional preprocessing.

**Fig. 5:**
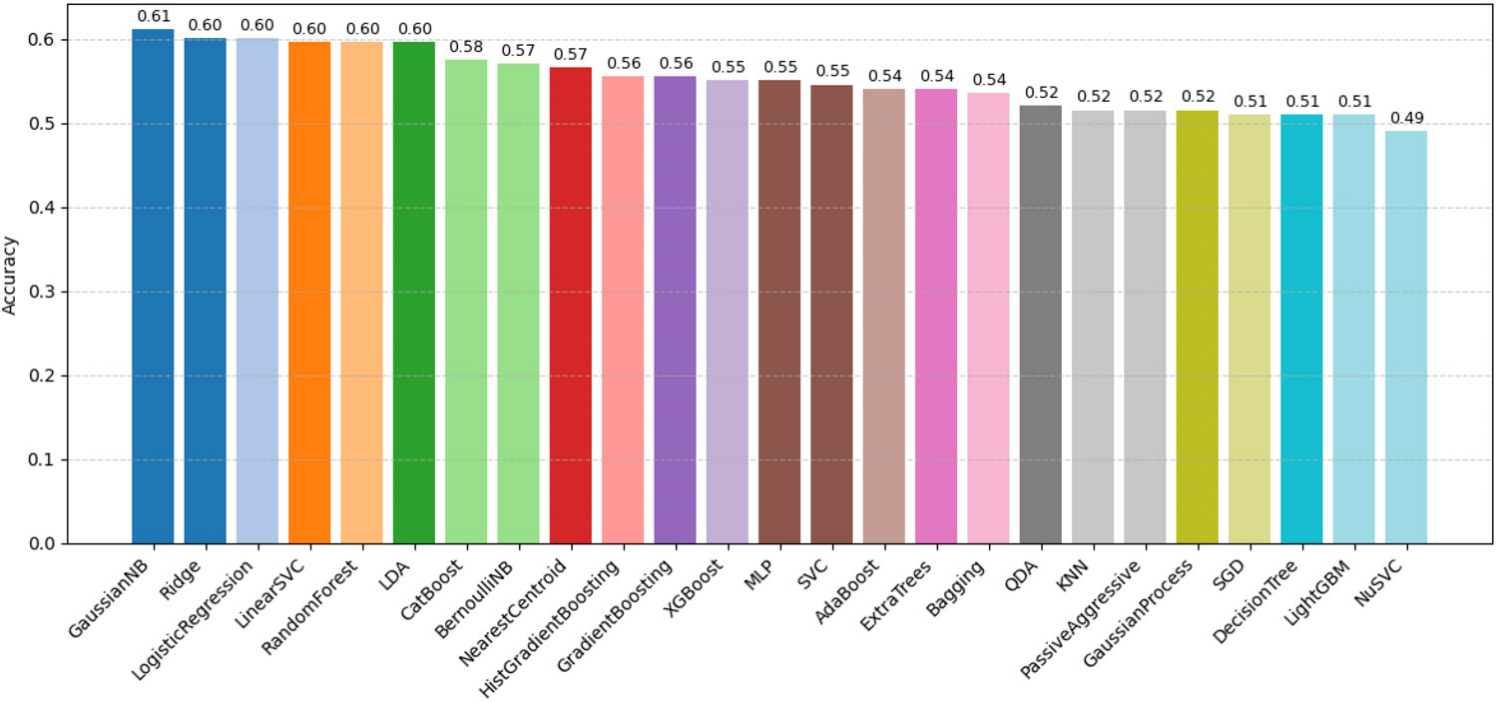
Accuracy comparison of all models.

**Fig. 6:**
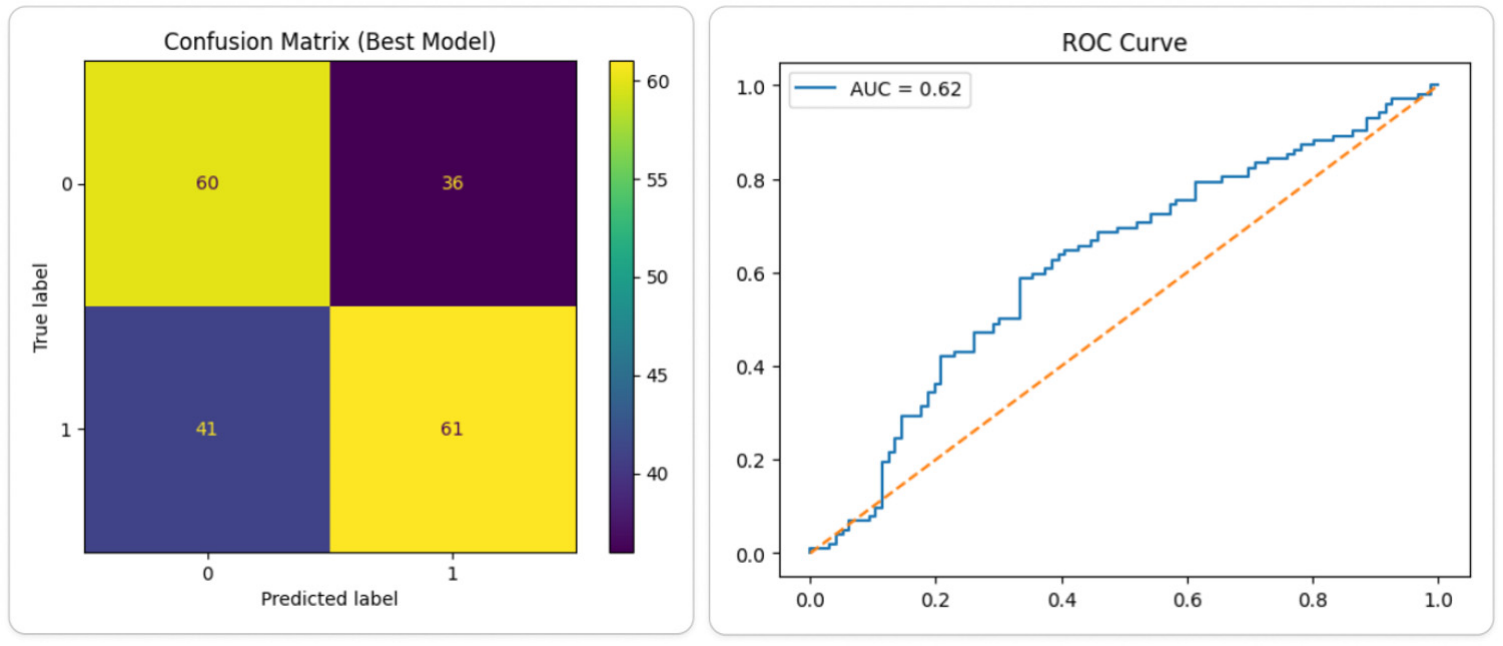
Confusion matrix and ROC for best performing model (GaussianNB).

To demonstrate the practical applicability of the BD-KDD dataset, a baseline machine learning experiment was conducted using multiple standard classifiers on the structured clinical features. The dataset was preprocessed using feature engineering (e.g., blood urea–creatinine ratio, electrolyte ratio, and hematological interactions), followed by median imputation and feature scaling. A total of 25 classifiers spanning linear, probabilistic, and tree-based were evaluated. Among them, Gaussian Naive Bayes achieved the highest performance with an accuracy of 61.11% and F1-score of 0.61, followed closely by Logistic Regression and Ridge Classifier (∼60%). These results indicate that relatively simple probabilistic and linear models can effectively capture the underlying patterns in the dataset. The moderate accuracy reflects the inherent complexity and overlapping nature of real-world clinical data, where clear separability between healthy and diseased cases is limited. To visually support this analysis, Fig. 5 presents a comparative bar chart of the all performing models based on accuracy, highlighting the relative effectiveness of different classifiers. Additionally, Fig. 6 illustrates the confusion matrix and ROC curve of the best-performing model, demonstrating its classification capability and discriminative performance.

## Limitations

Although the BD-KDD dataset provides a structured collection of clinical and laboratory measurements for renal health assessment, several limitations should be considered. First, the dataset was obtained from a single diagnostic center, which may introduce geographical or institutional bias in the recorded clinical measurements and patient characteristics. As a result, the dataset may not fully represent broader population variability across different healthcare facilities or regions. Second, the dataset was compiled retrospectively from routine clinical laboratory records. Consequently, the availability of variables depends on the tests requested during patient diagnosis, and some potentially relevant clinical indicators commonly used in nephrology may not be included. Third, although the dataset contains 988 patient records, which is larger than several commonly used renal datasets, the sample size remains limited compared with large-scale national or multi-center clinical databases.

## Ethics Statement

This study utilizes retrospective clinical data obtained from the electronic medical records of Popular Diagnostic Center, Savar Branch, Dhaka, Bangladesh. Formal authorization for data access and use for academic research purposes was granted by the responsible medical authority of the institution. The data were originally collected during routine clinical diagnostic procedures, where informed consent is obtained from patients as part of standard healthcare practice. For this study, only fully anonymized records were used, and all personally identifiable information (e.g., names, contact details, identification numbers) was removed prior to data processing. The study complies with the ethical principles outlined in the Declaration of Helsinki and adheres to standard data protection and privacy regulations for handling clinical data. As the study involves retrospective anonymized data, no additional ethical risk to participants is present.

## CRediT authorship contribution statement

**Muhammad Towfiqur Rahman:** Conceptualization, Formal analysis, Investigation, Resources, Data curation, Supervision. **Salma Akter:** Data curation, Resources, Data curation. **Md. Masudul Islam:** Software, Validation, Formal analysis, Data curation, Writing – original draft, Visualization. **Md. Shafiqul Islam:** Validation, Formal analysis, Resources, Writing – review & editing, Supervision.

## Data Availability

Dataverse.BD-KDD: A Clinical Dataset of Kidney Disease Diagnosis (Original data)
